# Effect of school lockdown due to the COVID-19 pandemic on screen time among adolescents in Hungary: a longitudinal analysis

**DOI:** 10.3389/fpubh.2023.1233024

**Published:** 2023-11-30

**Authors:** David Major, Vince Fazekas-Pongor, Katalin Pártos, Adam G. Tabák, Zoltan I. Ungvari, Dániel Eörsi, Dorottya Árva, András Terebessy

**Affiliations:** ^1^Department of Public Health, Faculty of Medicine, Semmelweis University, Budapest, Hungary; ^2^Department of Internal Medicine and Oncology, Faculty of Medicine, Semmelweis University, Budapest, Hungary; ^3^UCL Brain Sciences, University College London, London, United Kingdom; ^4^Oklahoma Center for Geroscience and Healthy Brain Aging, University of Oklahoma Health Sciences Center, Oklahoma City, OK, United States; ^5^Vascular Cognitive Impairment and Neurodegeneration Program, Department of Neurosurgery, University of Oklahoma Health Sciences Center, Oklahoma City, OK, United States; ^6^Peggy and Charles Stephenson Cancer Center, Oklahoma City, OK, United States; ^7^International Training Program in Geroscience, Doctoral School of Basic and Translational Medicine, Departments of Translational Medicine and Public Health, Semmelweis University, Budapest, Hungary; ^8^Department of Health Promotion Sciences, College of Public Health, University of Oklahoma Health Sciences Center, Oklahoma City, OK, United States; ^9^Doctoral School of Mental Health Sciences, Semmelweis University, Budapest, Hungary

**Keywords:** screen time, lockdown, COVID-19, adolescent, longitudinal studies, quarantine, school closure

## Abstract

**Introduction:**

Studies indicate that due to school lockdown during the Coronavirus Disease 2019 (COVID-19) pandemic, screen time increased more steeply than pre-pandemic years. The aim of our study was to examine changes in screen time and its components (screen time spent on videos, games, homework, and other activities) of adolescents affected by COVID-19 school closures compared to controls from pre-pandemic years and to assess the effect of family structure and family communication.

**Methods:**

Two sets of ninth-grader boys and girls transitioning into 10th grade were included in the analysis. The ‘pre-COVID classes’ (controls) completed the baseline survey in February 2018 and the follow-up survey in March 2019. ‘COVID classes’ (cases) completed the baseline survey in February 2020 (1 month before the COVID-19-related school lockdowns) and the follow-up survey in March 2021. Linear mixed models stratified by sex were built to assess the change in screen time over one year adjusted for family structure and communication.

**Results:**

Our study population consisted of 227 controls (128 girls, 99 boys) and 240 cases (118 girls, 122 boys). Without COVID-19, overall screen time did not change significantly for boys, but there was a decrease in screen time for gaming by 0.63 h, which was accompanied by an increase of 1.11 h in screen time for other activities (consisting mainly of social media and communication). Because of the pandemic, all components increased by 1.44–2.24 h in boys. Girls’ screen time and its components remained stable without school lockdown, while it increased for videos and homework by 1.66–2.10 h because of school lockdown. Living in a single-parent household was associated with higher, while better family communication resulted in lower screen time.

**Discussion:**

Our results indicate that COVID-19-related school lockdowns modified the age-specific increase in screen time for boys and girls as well. This trend, however, may be counterbalanced by improving communication between family members.

## 1 Introduction

During the Coronavirus Disease 2019 (COVID-19) pandemic, countries worldwide introduced different non-pharmaceutical measures to reduce and delay the surge of COVID-19 cases and mortality (1, 2). One such intervention was the initiation of school lockdowns and the provision of online education. As a result, face-to-face classes were replaced with online education from home, and families had to adapt abruptly to these drastically new circumstances (3).

Cross-sectional studies and their meta-analysis suggest that screen time was higher among students during the COVID-19 pandemic compared to pre-pandemic years (4, 5). However, cross-sectional studies suffer from limited power and are unable to differentiate between cohort and period effects. Thus, longitudinal studies examining changes before and during the COVID-19 pandemic are required to more precisely describe school closure-related changes in screen time. One such longitudinal study conducted among Canadian school-aged students, who were followed over multiple years, found a steady increase in screen time before the pandemic and a much steeper increase during COVID-19-related school closures, corroborating cross-sectional studies (6).

Screen time habits seem to show certain sex differences. For example, girls are more likely to be active on social media, while boys are more likely to engage in gaming (7). Increased screen time has been extensively associated with a wide variety of negative outcomes, such as obesity, inadequate amount of physical activity, poor sleep quality, depressive symptoms, suicidal thoughts, or not meeting certain developmental milestones (8–10).

Several determinants of screen time among adolescents are well described in the literature. Family structure seems to be an especially important factor related to screen time. Children in single-parent households and reconstituted families tend to have longer screen times (11). Communication between parents and their offspring may also play a key role, as certain communication styles are more successful than others in regulating the duration of screen time (12). Even though negative behaviors during adolescence may persist into adult life (13), certain factors, such as family, peer, or school support, may prevent the continuity of these unhealthy behaviors into adulthood (13).

Since screen time is associated with deleterious consequences on both physical and mental health of adolescents, it is extremely important to elucidate how pandemic-related factors, such as school lockdowns, modify screen time habits. Furthermore, as screen time changes with aging during adolescence (14), the best setting to investigate the effect of the pandemic-related school closures on screen time involves a longitudinal study design with a historical reference group unaffected by the pandemic. Thus, we aimed to examine school lockdown-related changes in overall screen time and its components (watching videos, playing games, doing homework, or other activities [e.g., social media]) by comparing two sets of ninth-grader boys and girls transitioning into 10th grade: one set not affected by COVID-19 and another affected by COVID-19 lockdowns. Furthermore, we also aimed to examine whether family structure or family communication modify the observed effect on screen time.

## 2 Materials and methods

### 2.1 Study design

The present study is a secondary analysis of data collected during a school-based health education program (Balassagyarmat Health Education Program [BEP]) that aimed to improve health literacy and focused on sexual health, substance use, basic life support, infection control, nutrition, and physical activity but did not cover screen time as a topic. The program was described in detail previously (15). In short, consecutive ninth graders were recruited in BEP from all five secondary schools (three grammar schools and two vocational secondary schools) in a northern Hungarian town (Balassagyarmat, approximately 15,000 inhabitants) in 2018–2020. Participants first completed a baseline survey, then participated in the health education program that spanned over a year. After completing the program, students filled in a follow-up survey, approximately one year after the baseline survey in 10th grade.

For the present analysis, we selected nineth-grade participants who completed their baseline assessment in either 2018 or 2020 (not 2019). For controls (baseline in 2018, unaffected by the COVID-19 pandemic), both baseline and follow-up surveys were filled in online under the supervision of research assistants (teachers were not present during the survey process) during school hours. For cases (baseline in 2020, affected by the COVID-19 pandemic during follow-up), baseline assessment took place in circumstances identical to controls, while the follow-up survey was filled in at home because of the school closures. Students were asked to complete the survey during specified school hours, and they could contact a research assistant online in case of any questions. Students recruited in 2019 were excluded from the current analysis because their follow-up survey was conducted during the early, transitional phase of school lockdown. The questionnaire and methodology for this study was approved by the Institutional Review Board of Semmelweis University (SE TUKEB: 276/2017). Parental approval of the participants was sought with an opt-out procedure.

### 2.2 Participants

**Figure 1:**
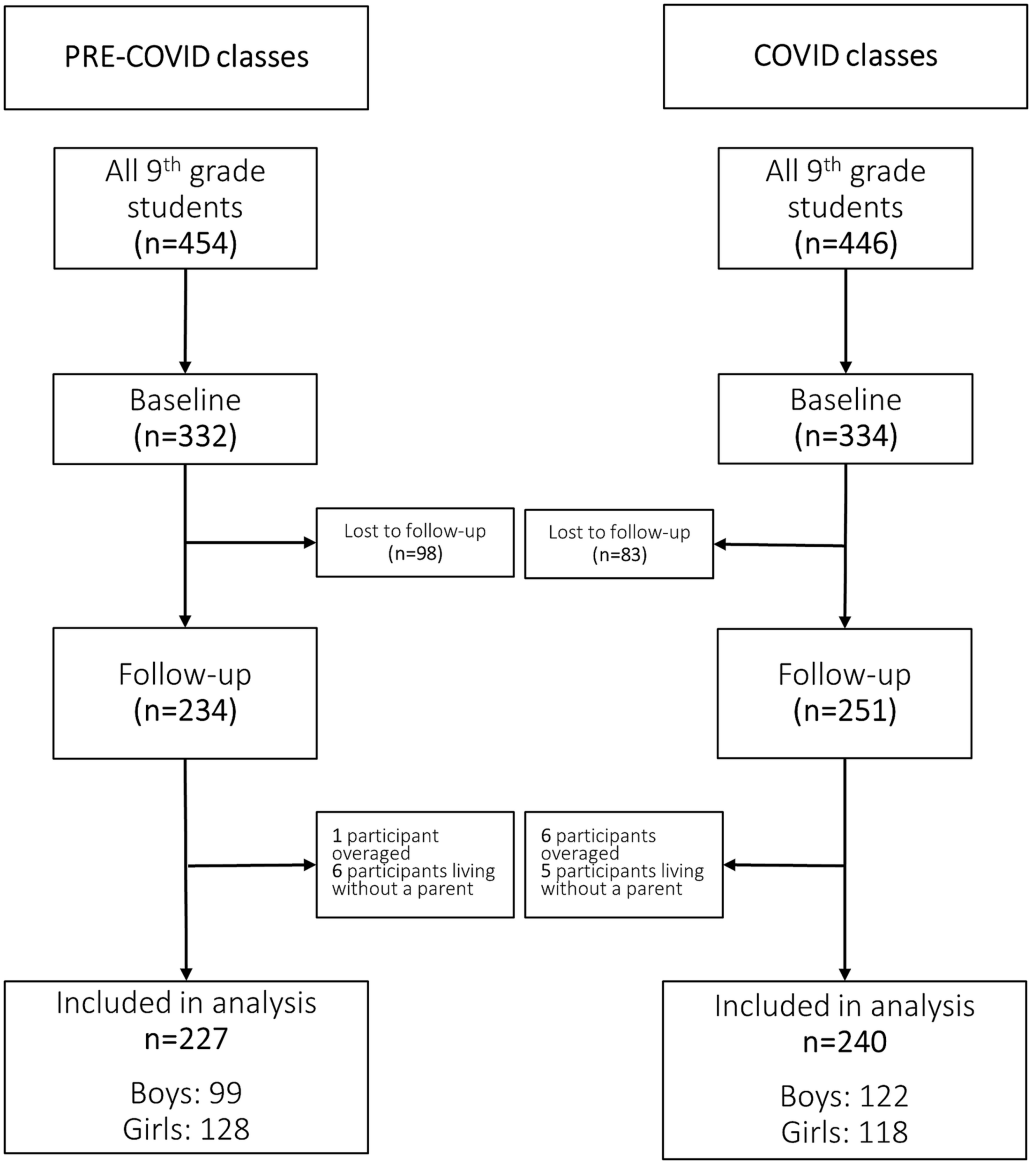
Flowchart of study participants.

All ninth-grader students were invited to participate. In 2018, out of the 454 ninth-grade students of the ‘pre-COVID classes’ (defined as controls), 332 agreed to participate at baseline resulting in a 0.73 sampling fraction. The baseline survey was completed in February 2018, while the follow-up survey was completed in March 2019. In the control group, 98 students were lost to follow-up. In 2020, out of the 446 ninth-grade students in the ‘COVID classes’ (defined as cases), 334 agreed to participate at baseline, resulting in a 0.75 sampling fraction. Cases completed the baseline survey in February 2020 (approximately 1 month before the COVID-19-related school lockdowns in Hungary) and the follow-up survey in March 2021. In the case group, 83 students were lost to follow-up. Flowchart of participants is presented in Figure 1. During the study period, school lockdowns affected students from 16 March 2020 to 2 June 2020 and then from 11 November 2020 to the end of study. According to the Organization for Economic Co-operation and Development (OECD) report, Hungarian secondary schools were fully closed for 164 days (excluding school holidays, public holidays and weekend) between 1 January 2020 and 20 May 2021 (16). Overaged students (mean age + 3 SD) and those living without a biological or stepparent were excluded.

### 2.3 Outcomes

To evaluate screen time, we used four questions based on the Hungarian version of the Health Behavior in School-aged Children study (HBSC 2014) (17, 18): (1) *‘How many hours a day, in your free time, do you usually spend watching TV, videos (including YouTube or similar services), DVDs, and other entertainment on a screen?’* (2) *‘How many hours a day, in your free time, do you usually spend playing games on a computer, games console, tablet (like iPad), smartphone or other electronic device (not including moving or fitness games)?’* (3) *‘How many hours a day, in your free time, do you usually spend using electronic devices such as computers, tablets (like iPad) or smart phones for other purposes, for example, homework, emailing, tweeting, Facebook, chatting, surfing the internet?’* (4) *‘How many hours of this do you spend on doing homework?’*. Students were required to express the amount of time spent on each activity as hours and fractions of an hour. The items resulted in five outcome variables: overall screen time (sum of Question 1, 2 and 3), screen time for watching videos (Question 1), playing games (Question 2), doing homework (Question 4), and screen time for other purposes (derived as the difference between data provided in Questions 3 and 4) representing time spent mainly on social media activities and communication).

### 2.4 Covariates

Since studies indicate that family support is an important protective factor against unhealthy behaviors among adolescents (13), we adjusted our results for family structure (living with two parents, with one parent, or in a stepfamily) and family communication [short version of the Clear Communication Scale from Family Dynamics Measure II [FDMII] (18, 19)] in our analyses. To assess family communication, we used the Hungarian version of the FDMII implemented in the Hungarian HSBC study 2014 (Cronbach-alfa: 0.74) (17). The questionnaire consists of four Likert items with a maximum score of 20. A higher score represents more positive judgement on family communication (17).

### 2.5 Statistical analysis

All analyses were conducted stratified by sex. Descriptive baseline data of pre-COVID vs. COVID classes and follow-up data of pre-COVID vs. COVID classes were compared with Chi-squared tests for categorical variables and independent samples *t*-tests for continuous variables. Descriptive baseline vs. follow-up data within pre-COVID and COVID classes were compared with marginal homogeneity tests for categorical variables and paired *t*-tests for continuous variables. Linear mixed models were built to assess the effect of COVID-19-related school lockdowns on overall screen time and on its individual components. *Model 1* includes case/control status as predictor, while Model 2 is adjusted for time-varying family structure and family communication. In our models, only family communication was treated as a continuous variable. To exclude the potential for a non-linear relationship, we tested whether adding a quadratic term of family communication would improve our models. Given that these quadratic terms were non-significant, we removed them from the final model to achieve parsimony. All other variables were categorical variables, and thus non-linearity was not investigated. Given that we had a sufficient number of cases (>200 individuals for each analysis) and only 2 time-points in a *random slope, random intercept* model, we decided to use the unstructured covariance matrix with the least number of assumptions, as it only increased the number of parameters in the model minimally (by one) compared to other frequently used covariance structures (e.g., variance component or autoregressive). Furthermore, we also tested the information criteria (AIC, BIC) of the above covariance structures, and the unstructured covariance structure had the lowest values. All statistical analyses were performed using IBM SPSS Statistics version 28.0.0.0. Statistical significance was set at *p* < 0.05.

## 3 Results

A total of 234 controls and 251 cases were eligible for our analysis. We excluded seven participants due to being overaged, and 11 students due to living without a parent. Thus, the final analytical sample consisted of 467 pupils: 227 pre-COVID controls (128 girls and 99 boys) and 240 cases affected by COVID-19 (118 girls and 122 boys) (Figure 1).

Few differences were present between pre-COVID and COVID classes. Female students were 0.22 years older in the COVID classes compared to pre-COVID classes. Furthermore, the family structure of girls in the pre-COVID and COVID groups was different at baseline: the proportion of two-parent families was lower in the COVID group. As for males, there were no differences between the pre-COVID and COVID groups in age, family structure, or family communication (Table 1).

**Table 1:** Descriptive statistics of pre-COVID and COVID classes.

	Pre-COVID classes		COVID classes	
	Baseline	Follow-up	Baseline	Follow-up
Boys				
*n*	99		122	
Age, mean ± SD	16.08 ± 0.60	–	16.16 ± 0.63	–
Family structure, *n* (%)				
Two-parent	71 (71.7%)^b^	66 (66.7%)^b^	85 (69.7%)	83 (68.0%)
Single-parent	17 (17.2%)^b^	19 (19.2%)^b^	20 (16.4%)	21 (17.2%)
Stepfamily	11 (11.1%)^b^	14 (14.1%)^b^	17 (13.9%)	18 (14.8%)
Family communication, mean ± SD	17.53 ± 2.61^b^	16.44 ± 3.56^b^	16.97 ± 3.26	16.79 ± 3.56
Girls				
*n*	128		118	
Age, mean ± SD	15.92 ± 0.68^a^	–	16.14 ± 0.64^a^	–
Family structure, *n* (%)				
Two-parent	94 (73.4%)^a^	92 (71.9%)	78 (66.1%)^a^	73 (61.9%)
Single-parent	25 (19.5%)^a^	26 (20.3%)	19 (16.1%)^a^	27 (22.9%)
Stepfamily	9 (7.0%)^a^	10 (7.8%)	21 (17.8%)^a^	18 (15.3%)
Family communication, mean ± SD	17.43 ± 2.56^b^	16.02 ± 4.15^b^	17.00 ± 3.11^b^	16.38 ± 3.84^b^

COVID: Coronavirus Diseases 2019; SD: standard deviation.

a *p* < 0.05 (Baseline data of pre-COVID vs. COVID classes and follow-up data of pre-COVID vs. COVID classes were compared with Chi-squared tests for categorical variables and independent samples *t*-tests for continuous variables).

b *p* < 0.05 (Baseline vs. follow-up data within pre-COVID and COVID classes were compared with Marginal Homogeneity tests for categorical variables and paired samples *t*-tests for continuous variables).

Similarly, we observed some changes over the one-year follow-up. The FDMII score significantly decreased in case and control girls during follow-up. As for boys, the FDMII score as well as the proportion of two-parent families decreased in controls during follow-up. We observed no similar change in cases (Table 1).

### 3.1 Changes in screen time of boys

**Figure 2:**
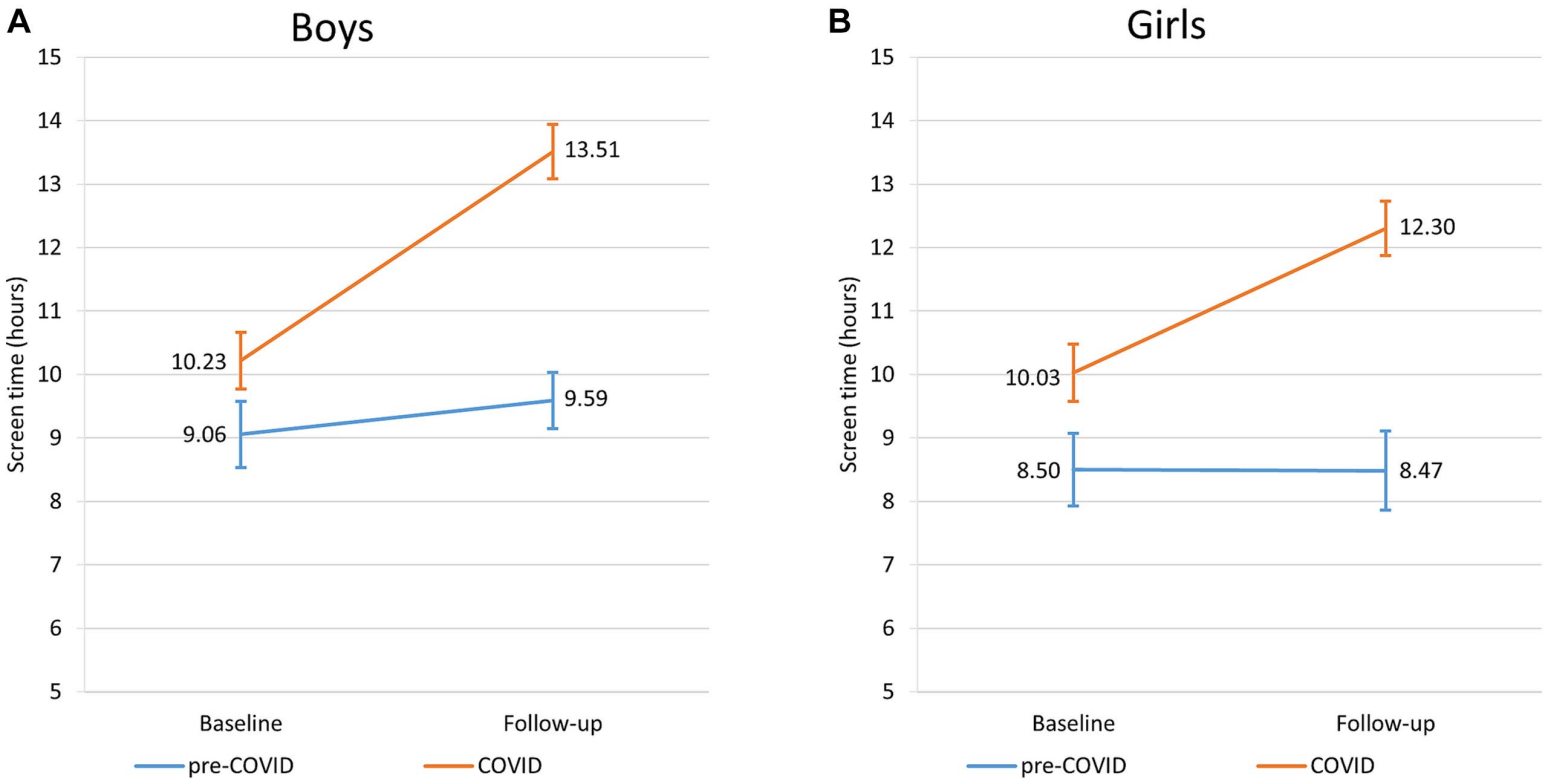
Changes of overall screen time of pre-COVID and COVID classes for boys **(A)** and girls **(B)**.

**Figure 3:**
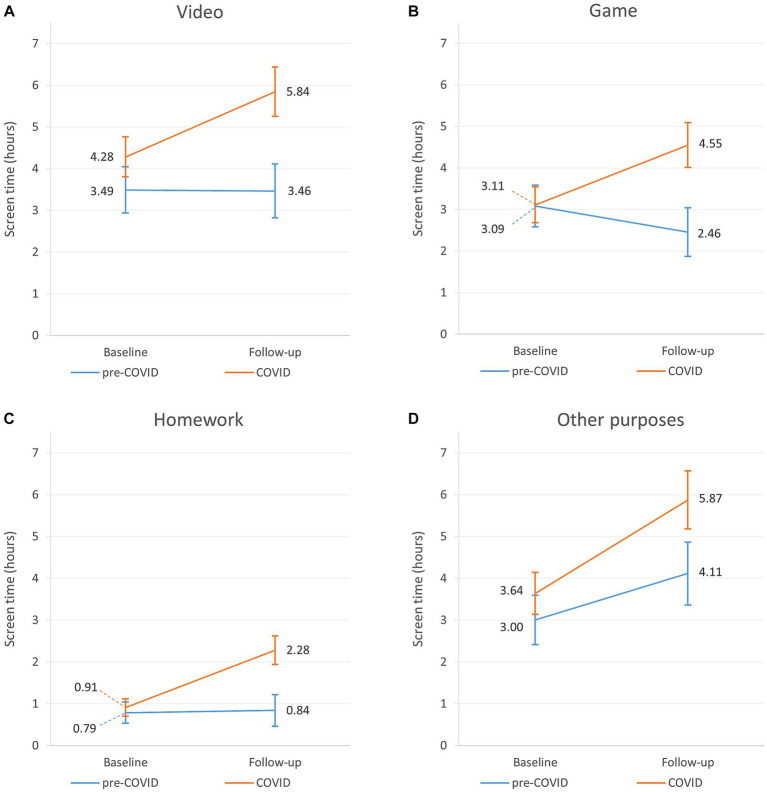
Changes of the individual components of screen time of pre-COVID and COVID classes for boys: screen time on watching videos **(A)**, playing games **(B)**, doing homework **(C)**, and other purposes **(D)**.

According to *Model 1,* cases’ overall screen time was higher by 1.35 (95% CI: 0.12–2.57) hours compared to controls. This difference became non-significant after adjustment for family structure and family communication (MD: 1.17 [−0.06–2.40]; 9.06 [95% CI: 8.03–10.08] vs. 10.23 [95% CI: 9.35–11.09] hours) (Table 2; Figure 2). As *Model 1* and *Model 2* yielded similar results for the individual components of screen time, we only provide detailed description of *Model 2* in the following. According to *Model 2*, at baseline cases spent 0.79 (95% CI: 0.13–1.45) hours more on watching videos compared to controls (3.49 [95% CI: 2.93–4.04] vs. 4.28 [95% CI: 3.80–4.76] hours), while screen time of controls and cases was similar for playing games (3.08 [95% CI: 2.59–3.58] vs. 3.11 [95% CI: 2.68–3.54] hours), doing homework (0.79 [95% CI: 0.53–1.04] vs. 0.91 [95% CI: 0.70–1.12] hours), and other purposes (3.00 [95% CI: 2.41–3.59] vs. 3.63 [95% CI: 3.13–4.14] hours) (Table 3; Figure 3).

**Table 2:** Results of liner mixed models for overall screen time expressed in hours.

	Model 1		Model 2^b^	
	Estimate (95% CI)	*p* value	Estimate (95% CI)	*p* value
Boys				
Intercept	8.46 (7.51–9.40)		8.35 (7.34–9.36)	
Classes				
pre-COVID	ref.			
COVID	**1.35 (0.12–2.57)**^**a**^	**0.032**	1.17 (−0.06–2.40)	0.063
pre-COVID^a^Time				
Baseline	ref.		ref.	
Follow-up	0.81 (−0.32–1.93)	0.158	0.53 (−0.59–1.66)	0.349
COVID^a^Time				
Baseline	ref.		ref.	
Follow-up	**3.27 (2.25–4.29)**^**a**^	**<0.001**	**3.29 (2.29–4.29)**^**a**^	**<0.001**
Family structure				
two-parent	–	–	ref.	
single-parent	–	–	0.66 (−0.69–2.01)	0.335
stepfamily	–	–	1.45 (−0.02–2.92)	0.053
Family communication	–	–	−0.78 (−1.32 – −0.24)*	**0.005**
Girls				
Intercept	8.31 (7.55–9.07)		8.18 (7.37–8.99)	
Classes				
pre-COVID	ref.		ref.	
COVID	1.52 (0.43–2.60)*	**0.007**	**1.53 (0.44–2.62)**^**a**^	**0.006**
pre-COVID^a^Time				
Baseline	ref.		ref.	
Follow-up	0.20 (−0.63–1.05)	0.646	−0.02 (−0.88–0.84)	0.969
COVID^a^Time				
Baseline	ref.		ref.	
Follow-up	**2.47 (1.57–3.38)**^**a**^	**<0.001**	**2.27 (1.37–3.18)**^**a**^	**<0.001**
Family structure				
two-parent	–	–	ref.	
single-parent	–	–	**1.16 (0.02–2.31)**^**a**^	**0.046**
stepfamily	–	–	−0.19 (−1.68–1.30)	0.804
Family communication	–	–	**−0.64 (−1.11 – −0.16)**^**a**^	**0.009**

95% CI: 95% Confidence Interval; COVID: Coronavirus Disease 2019. Bold values indicate significant results.

a *p* < 0.05.

b Adjusted for time-varying family structure and family communication.

**Table 3:** Results of liner mixed models for the individual components of boys’ screen time expressed in hours.

	Video				Game				Homework				Other purposes			
	Model 1		Model 2^b^		Model 1		Model 2^b^		Model 1		Model 2^b^		Model 1		Model 2^b^	
	Estimate (95% CI)	value of p	Estimate (95% CI)	value of p	Estimate (95% CI)	value of p	Estimate (95% CI)	value of p	Estimate (95% CI)	value of p	Estimate (95% CI)	value of p	Estimate (95% CI)	value of p	Estimate (95% CI)	value of p
Intercept	3.24 (2.74–3.75)		3.10 (2.56–3.65)		2.69 (2.24–3.15)		2.58 (2.09–3.06)		0.79 (0.56–1.01)		0.77 (0.52–1.03)		2.79 (2.26–3.33)		2.71 (2.12–3.30)	
Classes																
pre-COVID	ref.		ref.		ref.		ref.		ref.		ref.		ref.		ref.	
COVID	**0.83 (0.16–1.49)**^**a**^	**0.015**	**0.79 (0.13–1.45)**^**a**^	**0.019**	0.12 (−0.48–0.72)	0.685	0.03 (−0.57–0.62)	0.931	0.12 (−0.17–0.42)	0.413	0.12 (−0.18–0.42)	0.425	0.68 (−0.02–1.38)	0.055	0.63 (−0.08–1.34)	0.081
pre-COVID^a^Time																
Baseline	ref.		ref.		ref.		ref.		ref.		ref.		ref.		ref.	
Follow-up	0.07 (−0.52–0.67)	0.811	−0.03 (−0.63–0.58)	0.930	−0.46 (−0.95–0.03)	0.066	**−0.63 (−1.12 – −0.14)**^**a**^	**0.012**	0.05 (−0.36–0.45)	0.827	0.05 (−0.36–0.47)	0.800	**1.18 (0.33–2.04)**^**a**^	**0.007**	**1.11 (0.25–1.97)**^**a**^	**0.012**
COVID^a^Time																
Baseline	ref.		ref.		ref.		ref.		ref.		ref.		ref.		ref.	
Follow-up	**1.59 (1.07–2.10)**	**<0.001**	**1.56 (1.05–2.08)**^**a**^	**<0.001**	**1.47 (1.05–1.89)**^**a**^	**<0.001**	**1.44 (1.02–1.85)**^**a**^	**<0.001**	**1.37 (1.02–1.73)**^**a**^	**<0.001**	**1.38 (1.02–1.73)**^**a**^	**<0.001**	**2.25 (1.50–3.00)**^**a**^	**<0.001**	**2.24 (1.49–2.99)**^**a**^	**<0.001**
Family structure																
two-parent	–	–	ref.		–	–	ref.		–	–	ref.		–	–	ref.	
single-parent	–	–	**0.94 (0.18–1.70)**^**a**^	**0.015**	–	–	**0.78 (0.10–1.46)**^**a**^	**0.025**	–	–	0.13 (−0.22–0.48)	0.479	–	–	0.52 (−0.27–1.30)	0.197
stepfamily	–	–	0.22 (−0.63–1.07)	0.608	–	–	0.75 (−0.02–1.52)	0.057	–	–	−0.09 (−0.47–0.29)	0.635	–	–	0.37 (−0.50–1.24)	0.404
Family communication	–	–	**−0.32 (−0.62 – −0.02)**^**a**^	**0.040**	–	–	**−0.41 (−0.66 – −0.15)**^**a**^	**0.002**	–	–	0.10 (−0.06–0.26)	0.223	–	–	−0.07 (−0.42–0.29)	0.722

95% CI: 95% Confidence Interval; COVID: Coronavirus Disease 2019. Bold values indicate significant results.

a *p* < 0.05.

b Adjusted for family structure and family communication.

In controls, with aging overall screen time did not show a significant change (9.06 [95% CI: 8.03–10.08] vs. 9.59 [95% CI: 8.55–10.63] hours) (Figure 2), however the pattern of its components was altered. Screen time for watching videos remained stable (3.49 [95% CI: 2.93–4.04] vs. 3.46 [95% CI: 2.81–4.11] hours), playing games decreased by 0.63 (95% CI: −1.12 – −0.14) hours (3.08 [95% CI: 2.59–3.59] vs. 2.45 [95% CI: 1.87–3.04] hours), doing homework also remained stable (0.79 [95% CI: 0.53–1.04] vs. 0.84 [95% CI: 0.46–1.22] hour), while screen time for other purposes increased by 1.11 (95% CI: 0.25–1.97) hours (3.00 [95% CI: 2.41–3.59] vs. 4.11 [95% CI: 3.36–4.87] hours) (Table 3; Figure 3).

In the COVID classes overall screen time increased by 3.29 (95% CI: 2.29–4.29) hours (10.23 [95% CI: 9.35–11.09] vs. 13.51 [95% CI: 12.49–14.53] hours) during the follow-up period (Table 2; Figure 2). All components of screen time also increased. Screen time spent on watching videos increased by 1.56 (95% CI: 1.05–2.08) hours (4.28 [95% CI: 3.80–4.76] vs. 5.84 [95% CI: 5.25–6.44] hours), playing games – as opposed to controls – increased by 1.44 (95% CI: 1.02–1.85) hours (3.11 [95% CI: 2.68–3.54] vs. 4.55 [95% CI: 4.01–5.09] hours), homework increased by 1.38 (95% CI: 1.02–1.73) hours (0.91 [95% CI: 0.70–1.12] vs. 2.29 [95% CI: 1.94–2.62] hours), and other purposes increased by 2.24 (95% CI: 1.49–2.99) hours (3.63 [95% CI: 3.13–4.14] vs. 5.87 [95% CI: 5.18–6.56] hours) (Table 3; Figure 3).

Furthermore, we found that boys living with only one parent spent more screen time on watching videos and playing games. We also found that boys who scored higher on family communication had lower overall screen time and screen time for watching videos and playing games (Table 2; Table 3).

### 3.2 Changes in screen time of girls

**Figure 4:**
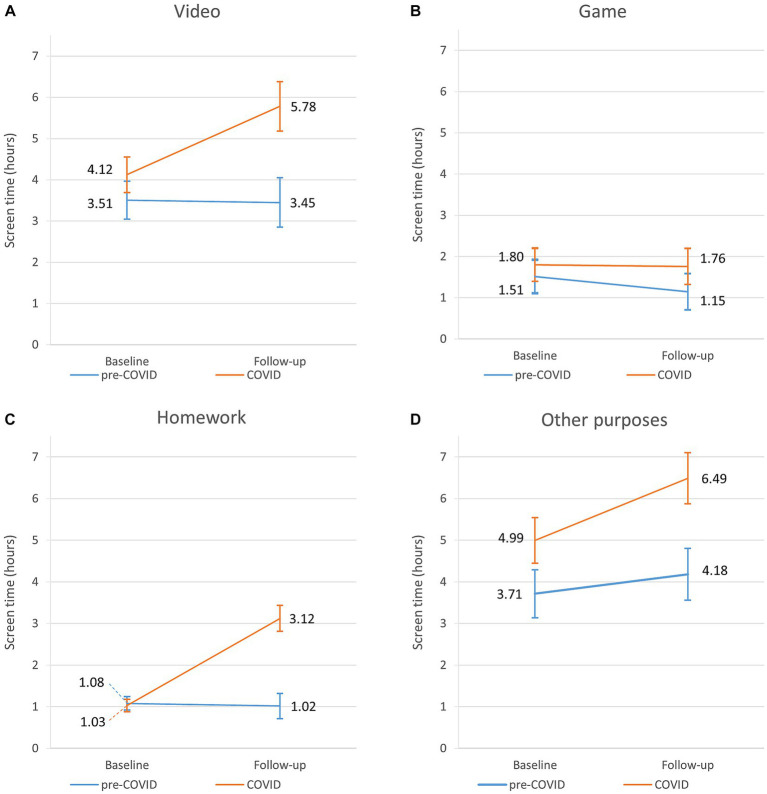
Changes of the individual components of screen time of pre-COVID and COVID classes for girls: screen time on watching videos **(A)**, playing games **(B)**, doing homework **(C)**, and other purposes **(D)**.

Similarly to boys, *Model 1* and *Model 2* yielded similar results in girls. According to *Model 2*, we detected that cases’ overall screen time at baseline was higher by 1.53 (0.44–2.62) hours than that of controls (8.50 [95% CI: 7.62–9.38] vs. 10.03 [95% CI: 9.19–10.87] hours) (Table 2; Figure 2). As for the individual components, screen time spent on watching videos was higher in cases by 0.62 (95% CI: 0.06–1.17) hours than in controls (3.51 [95% CI: 3.05–3.96] vs. 4.13 [95% CI: 3.69–4.55] hours], there was no significant difference in screen time for playing games (1.51 [95% CI: 1.10–1.93] vs. 1.79 [95% CI: 1.40–2.19] hours) and doing homework (1.08 [95% CI: 0.92–1.24] vs. 1.03 [95% CI: 0.88–1.17] hours), but screen time for other purposes was also higher by 1.28 (95% CI: 0.57–2.00) hours in cases compared to controls (3.71 [95% CI: 3.14–4.29] vs. 4.99 [95% CI: 4.45–5.54] hours) (Table 4; Figure 4).

**Table 4:** Results of liner mixed models for the individual components of girls’ screen time expressed in hours.

	Video				Game				Homework				Other purposes			
	Model 1		Model 2^b^		Model 1		Model 2^b^		Model 1		Model 2^b^		Model 1		Model 2^b^	
	Estimate (95% CI)	value of p	Estimate (95% CI)	value of p	Estimate (95% CI)	value of p	Estimate (95% CI)	value of p	Estimate (95% CI)	value of p	Estimate (95% CI)	value of p	Estimate (95% CI)	value of p	Estimate (95% CI)	value of p
Intercept	3.38 (2.99–3.76)		3.35 (2.93–3.76)		1.54 (1.18–1.90)		1.63 (1.25–2.02)		1.05 (0.92–1.18)		1.04 (0.90–1.19)		3.48 (2.97–3.99)		3.25 (2.72–3.78)	
Classes																
pre-COVID	ref.		ref.		ref.		ref.		ref.		ref.		ref.		ref.	
COVID	**0.65 (0.10–1.20)**^**a**^	**0.021**	**0.62 (0.06–1.17)**^**a**^	**0.030**	0.31 (−0.20–0.82)	0.231	0.28 (−0.24–0.80)	0.288	−0.04 (−0.23–0.14)	0.633	−0.05 (−0.25–0.14)	0.593	**1.27 (0.54–2.00)**^**a**^	**<0.001**	**1.28 (0.57–2.00)**^**a**^	**<0.001**
pre-COVID^a^Time																
Baseline	ref.		ref.		ref.		ref.		ref.		ref.		ref.		ref.	
Follow-up	0.05 (−0.46–0.56)	0.860	−0.06 (−0.58–0.46)	0.834	−0.31 (−0.76–0.15)	0.183	−0.37 (−0.82–0.08)	0.110	−0.06 (−0.33–0.21)	0.651	−0.06 (−0.36–0.23)	0.677	0.52 (−0.08–1.13)	0.090	0.47 (−0.15–1.08)	0.135
COVID^a^Time																
Baseline	ref.		ref.		ref.		ref.		ref.		ref.		ref.		ref.	
Follow-up	**1.73 (1.20–2.26)**^**a**^	**<0.001**	**1.66 (1.13–2.19)**^**a**^	**<0.001**	−0.03 (−0.49–0.43)	0.889	−0.04 (−0.50–0.42)	0.862	**2.10 (1.82–2.38)**^**a**^	**<0.001**	**2.10 (1.80–2.40)**^**a**^	**<0.001**	**1.59 (0.97–2.21)**^**a**^	**<0.001**	**1.49 (0.87–2.12)**^**a**^	**<0.001**
Family structure																
two-parent	–	–	ref.		–	–	ref.		–	–	ref.		–	–	ref.	
single-parent	–	–	0.33 (−0.31–0.97)	0.310	–	–	−0.23 (−0.77–0.31)	0.399	–	–	0.02 (−0.21–0.26)	0.842	–	–	**0.92 (0.16–1.67)**^**a**^	**0.017**
stepfamily	–	–	0.14 (−0.67–0.96)	0.729	–	–	−0.12 (−0.81–0.56)	0.720	–	–	0.08 (−0.21–0.37)	0.589	–	–	0.47 (−0.49–1.42)	0.338
Family communication	–	–	−0.27 (−0.55–0.01)	0.059	–	–	−0.12 (−0.35–0.11)	0.320	–	–	0.01 (−0.11–0.12)	0.972	–	–	−0.29 (−0.61 – −0.03)	0.080

95% CI: 95% Confidence Interval; COVID: Coronavirus Disease 2019. Bold values indicate significant results.

a *p* < 0.05.

b Adjusted for family structure and family communication.

During follow-up, control girls’ overall screen time remained stable (8.50 [95% CI: 7.62–9.38] vs. 8.47 [95% CI: 7.56–9.41] hours) (Table 2; Figure 2). Unlike boys’, control girls’ screen time did not show any significant change in the individual components with aging. They spent 3.45 h (95% CI: 2.85–4.04) on watching videos, 1.14 h (95% CI: 0.70–1.59) on playing games, 1.02 h (95% CI: 0.71–1.32) on doing homework and 4.18 h (95% CI: 3.56–4.80) for other screen time purposes (Table 4; Figure 4).

On the other hand, classes affected by COVID increased their overall screen time by 2.27 (95% CI: 1.37–3.18) hours (10.03 [95% CI: 9.19–10.87] vs. 12.30 [95% CI: 11.35–13.25] hours) (Table 2; Figure 2). As for the individual components, time spent on watching videos increased by 1.66 (95% CI: 1.13–2.19) hours (4.13 [95% CI: 3.69–4.55] vs. 5.79 [95% CI: 5.18–6.37] hours), playing games remained stable (1.80 [95% CI: 1.40–2.19] vs. 1.76 [95% CI: 1.32–2.19] hours), doing homework increased by 2.10 (95% CI: 1.80–2.40) hours (1.03 [95% CI: 0.88–1.17] vs. 3.13 [95% CI: 2.81–3.43] hours), and screen time spent on other activities increased by 1.49 (95% CI: 0.87–2.12) hours (4.99 [95% CI: 4.45–5.54] vs. 6.48 [95% CI: 5.87–7.10] hours) during follow-up (Table 4; Figure 4).

Living in a single-parent household was associated with higher overall screen time and screen time spent on social media. Higher score on family communication was associated with lower overall screen time in girls, however, it showed no association with the individual components of screen time (Table 2, 4).

## 4 Discussion

Our study offers insight into adolescents’ screen time habits before the COVID-19 pandemic and how it changed due to school lockdown. In our sample, overall screen time was around 9–10 h at baseline which is markedly higher than the recommendations (20). Both control and case boys spent the most screen time on watching videos at baseline. Without the presence of lockdown, aging of boys was associated with an increase in screen time for other purposes and a decrease in that for playing games, but the overall screen time remained stable. As for girls, the highest screen time was measured for other purposes – which consisted of mainly social media and communication – at baseline, and aging had no significant effect on their overall screen time and screen time habits. Based on our results, the COVID-19 pandemic modified these age-related trends. Boys affected by lockdown increased their overall screen time and screen time in every examined activity among which screen time for other activities showed the greatest increase. During the COVID-19 pandemic, girls also increased their overall screen time and its individual components, except for screen time for playing games, which did not change. As for family-related variables, we found that living with only one parent was associated with higher screen time for watching videos and playing games for boys and for overall screen time and social media for girls. Better family communication resulted in lower screen time for watching videos and playing games for boys and with overall screen time for girls. However, these family-related variables had no effect on the overall direction and size of the observed associations.

Several studies have reported that adolescents’ screen time had been higher than the recommended amount of two hours/day recreational screen time for children and adolescents (5–17 years) (20) even before the COVID-19 pandemic and that it increased significantly during lockdown (6). Two recent meta-analyses found that total daily screen time of adolescents increased by around 0.9–1.8 h/day during the COVID-19 pandemic (5, 21), which is lower than our results of 3.3 h increase for boys and 2.3 h for girls. Even though screen time tends to increase with age in adolescents (14, 22, 23), this is not supported by our results. It must be noted, however, that our study examined changes in screen time over a relatively shorter period. On the other hand, our results do corroborate the few longitudinal studies that found that the increase was much more substantial during the pandemic compared to the pandemic-free period (6, 24). Furthermore, we extend previous observations by the finding that except for time spent on gaming in girls, all forms of screen time increased significantly both in girls and boys during the pandemic. In our sample, all kinds of device use (except for homework) already exceeded the recommendations at baseline, and the lockdown added a further 1–2 h to each dimension. This is alarming as higher screen time is associated to several negative outcomes on physical health (high blood pressure, obesity, low HDL cholesterol, disrupted stress regulation, insulin resistance, impaired vision, lower bone density, poor sleep) and mental health (depression, suicidal thoughts, electronic devices dependency, antisocial behavior) (9).

The experienced negative effects may differ by the type of activity adolescents pursue on electronic devices. In our study, boys were more engaged in playing games, while girls spent more screen time for other activities, which consisted of social media and communication activities in our case. These are parallel with the results of other studies (25–27). Furthermore, even the same type of screen time could have different effects on boys and girls. Girls, for instance, are more likely to develop symptoms of depression, anxiety, loneliness, and physical symptoms, such as headache and stomachache than boys as a consequence of social media use (28, 29). Moreover, social media use may also have a detrimental effect on the overall wellbeing of girls and may also predispose girls to the development of negative body image (28, 30–33). It has also been suggested that the risk of mental health problems increases at a lower threshold of screen time (two hours/day) in girls compared to boys (five hours/day) (34). This difference may be explained by the fact that girls are more concerned about social comparison, feedback, being accepted, and having intimate friendships (30, 35, 36).

In contrast to girls, boys spent more time on playing games in our study, which has been linked to unwanted consequences, such as depressive symptoms and lower life satisfaction (34, 37). These effects are more likely to appear after excessive amounts of gaming (34). A study found that anxiety-like symptoms appear after six or more hours of gaming, and this effect was observed only in boys, not in girls (38). This further supports that adolescents of different sexes react differently to different types of screen time. This is corroborated by a study that found greater activation in the medial frontal gyrus, the bilateral middle temporal gyri, and thalamic regions of men compared to women after gaming (37). This may explain why men are more likely to develop craving-like symptoms as a result of gaming and why they are more prone to develop gaming disorders (37). A study conducted on male internet gamers found that the reasons for gaming are entertainment, getting along with friends, stress relief, and habitual gaming. This study also found that habitual gamers are more likely to develop gaming disorder, indicating that apart from sex the reason for gaming also influences the appearance of disorders (39).

In our study, we found that adolescent boys living in single-parent households spend more time watching videos and play more games, while adolescent girls in single-parent households spend more time on social media. This is corroborated by another study that found that youth in single-parent households are more likely to exhibit unfavorable patterns of physical activity, participation in sports, and screen time behaviors (11, 40). Possible explanations to this may be the lack of time of single parents, which may be barrier for imposing restrictions on screen time, and the lack of financial resources to involve their children in extracurricular activities (11). This increased screen time, however, may be counterbalanced (at least in boys) by better communication, as seen in our study. This is in line with the results of a study that found that autonomy-supportive style of communication is more successful than controlling styles of communication, which was associated to increased screen time of children (12). Autonomy-supportive parenting aims to encourage the child’s volitional functioning by allowing choice and offering relevant rationale tailored to the child’s specific situation when introducing rules (12). Controlling style of communication on the other hand tries to impose the parent’s own will on the child and requires the child to feel and act in a certain way (12). These results emphasize that the communication style is a key factor for parents, who want to achieve changes in their child’s particular behavior, such as screen time.

### 4.1 Strengths and limitations

A major strength of our study is its longitudinal design, which enabled us to observe within participant trends of screen time. Studies indicate that longitudinal studies have much more statistical power and thus result in a more precise assessment of screen time, as shown by a study in which retrospective assessment was compared to longitudinal assessment (21). As observed by the authors, retrospective assessment tended to overestimate the true amount of screen time (21). Furthermore, our design allowed us to differentiate and compare the effect of aging and the pandemic. Another strength is that we examined the different dimensions of screen time, and thus were able to analyze how engagement in different screen time categories changed as a result of lockdown. A subsequent strength is that both case and control students came from the same settlement, and controls preceded cases by only two years reducing the potential of time period effects. Moreover, our study is one of the first to offer a deeper insight in screen time in Balassagyarmat, a typical city of a deprived region in central Europe.

A limitation of our study is that answers of student were not cross-referenced by parents. A study similar to ours found, however, that the estimation of screen time by students tends to be similar to the assessment of parents (6). Another limitation of our study is that we were not able to account for double screen time, for instance watching TV while scrolling social media. Our questions also did not focus exclusively on social media and had to be calculated *post hoc* from the HBSC 2014 questions. We also experienced baseline difference in males’ screen time on watching videos and females’ screen time on watching videos and other activities, which may be explained by the different time periods of the baseline and follow-up surveys (February vs. March) with different weather patterns and daylight hours. This, however, is less likely to have severely biased the observed trends between baseline and follow-up. Finally, our study did not contain information on other sedentary behaviors and level of physical activity.

## 5 Conclusion

In our study, we observed that during the study period screen time remained stable without the COVID-19 pandemic, but it increased as a result of school lockdown. With aging, in pandemic-free circumstances, girls’ screen time habits remained consistent, while boys decreased their time spent on games and caught up to girls in their time spent on social media and communication activities. Lockdown modified these trends. All individual components increased in both sexes, except for playing games for girls, which remained stable. As increased screen time may result in detrimental effects on physical and mental health, it is crucial to identify risk and protective factors that may influence the amount of screen time. We found, for instance, that children in single-parent households exhibited higher screen time, which may be attributed to the lack of time and financial resources of single parents. This, however, can be counterbalanced by better family communication, especially for males, who exhibited less time watching videos and playing games as a result of better family communication in our study. Public health programs implemented to decrease screen time should target families as a whole and promote improved family communication instead of solely focusing on adolescents. These interventions should help parents familiarize themselves with communication styles, as communication in itself does not necessarily lead to decreased screen time. It must be noted, however, that our observation related to family communication may be confounded by reverse causation, as more screen time may also result in worse communication between family members. Finally, since online homework is becoming more wide-spread, schools should also try to come up with assignments that require physically active involvement of students instead of passive activities, such as watching videos, which may further increase passive screen time of students unnecessarily.

## Data availability statement

The raw data supporting the conclusions of this article will be made available by the authors, without undue reservation.

## Ethics statement

The studies involving humans were approved by Institutional Review Board of Semmelweis University. The studies were conducted in accordance with the local legislation and institutional requirements. Written informed consent for participation in this study was provided by the participants' legal guardians/next of kin.

## Author contributions

DM, DE, DÁ, and ZU contributed to the conceptualization of the study. VF-P, AdT, and DM performed the statistical analysis. KP, VF-P, and DM wrote the first draft of the manuscript. AnT and AdT supervised the study. All authors contributed to the article and approved the submitted version.
